# Exploring Student Perceptions of Generative AI Therapists and Their Benefits and Challenges in Schools

**DOI:** 10.1002/ijop.70207

**Published:** 2026-03-30

**Authors:** Cecilia K. Y. Chan, Samson Tse

**Affiliations:** ^1^ Teaching and Learning Innovation Centre, Faculty of Education The University of Hong Kong Pok Fu Lam Hong Kong; ^2^ Department of Social Work and Social Administration, Centre of Development and Resources for Students The University of Hong Kong Pok Fu Lam Hong Kong

**Keywords:** AI literacy, generative AI (GenAI), mental health support, student perceptions, therapeutic misconception

## Abstract

This study investigates secondary school students' perceptions of generative AI (GenAI) as virtual mental health therapists within educational settings. Leveraging data from the written reflections of 69 Hong Kong secondary school students engaged in an AI literacy programme, the study applied inductive thematic analysis to explore perceived potentials and limitations of GenAI therapy chatbots. Students valued the accessibility, anonymity and cost‐effectiveness of AI‐powered therapy. However, significant concerns arose regarding the technology's lack of genuine empathy, limited understanding of non‐verbal cues and potential for inaccurate responses. Ethical issues, such as data privacy and trust, were highlighted, with students expressing warranted distrust regarding personal data handling. The findings emphasised the risk of over‐reliance on AI and the therapeutic misconception that AI generates more suitable advice. While students acknowledged GenAI as a supplementary tool for initial support, they underscored the irreplaceable role of human therapists for deep, personalised care. This research calls for comprehensive AI literacy education to help students effectively navigate the strengths and boundaries of AI in mental health support.

## 1 Introduction

Artificial intelligence (AI) is increasingly integrated into healthcare, including mental health, where AI interventions are now being utilised to supplement or partially replace in‐person services (Sweeney et al. 2021). However, concerns remain about whether AI‐assisted care can consistently provide better outcomes and genuinely empower users (Zidaru et al. 2021); moreover, there is limited research on how well service users accept and benefit from such tools that mimic human interaction (Fiske et al. 2019). Challenges such as data ethics, regulatory gaps, potential misuse and the risk of AI tools completely replacing traditional modes of care also need to be addressed (Hamdoun et al. 2023).

While there are some studies on the use of generative AI (GenAI) for mental health support in educational settings (Labadze et al. 2023), its application in secondary school environments remains underexplored. Studying GenAI therapies in secondary school is crucial, as young students may be more vulnerable to the risks of AI technologies. A solid understanding of the topic empowers them to make informed decisions about technology and its impact on their lives. This study addresses this gap by engaging secondary school students who have both theoretical and practical experience with GenAI to provide clearer insights into how GenAI can be effectively used as a mental health support tool in school settings. Using empirical data gathered from these students, this study seeks to bridge current research gaps and establish a more comprehensive understanding of GenAI's capabilities and limitations in this domain. Thus, this study addresses the following research questions:

1. How do secondary school students perceive the use of GenAI chatbots as mental health support tools in educational settings?
2. What benefits and challenges do students identify when engaging with AI‐powered therapy chatbots?
3. How might students' perceptions reflect gaps in AI literacy and understanding of AI's emotional capabilities?

## 2 Literature

### 2.1 Overview of GenAI in Education and Health Care

In education, GenAI offers interactions that closely resemble natural human‐to‐human conversations (Chan and Hu 2023) which can provide context‐aware, varied and empathetic responses to enhance user engagement. Its multimodal capabilities to handle text, images and voice recognition—as well as doing so in different languages (Chan and Colloton 2024) support diverse learning preferences, enhancing inclusivity. In addition, the scalability of GenAI makes it suitable for delivering education and support at scale for both small and large settings, supporting tasks from classroom queries to complex grading.

However, GenAI still poses significant challenges. Over‐reliance on AI can hinder students' development of critical thinking, creative thinking and problem‐solving skills (Chan and Hu 2023; Chan and Lee 2023). Its dependence on data patterns can lead to inaccuracies and potential misunderstandings (Chan and Colloton 2024), and GenAI's inability to grasp subtleties of learning may contribute to a superficial understanding of complex topics. Another issue concerns the impersonal nature of AI‐driven education, which makes it devoid of relational learning experiences inherent in human teaching (Chan and Lee 2023). Data privacy and security issues, including potential misuse and breaches of student information, further complicate its use. Addressing these weaknesses is essential for an effective and sustainable integration of GenAI that meets educational purposes. This echoes Chustecki's (2024) review on AI in health care; she states that AI offers significant benefits in health care, including more accurate diagnoses, personalised treatments, efficient resource allocation and reduced costs through early detection and administrative automation. However, it also poses notable risks such as algorithmic biases that perpetuate disparities, lack of transparency in decision‐making, patient data privacy concerns and potential safety issues in clinical implementation.

### 2.2 Current Applications of AI in School Mental Health Support

AI's potential to provide mental health support—including emotional support, stress management and counselling—within school settings is vast. For example, facial recognition technology can be used to recognise and assess students' emotional expressions to provide real‐time insights into their moods or mental health state (Simcock et al. 2020), enabling educators to modify their teaching approaches or provide additional support. Some AI applications can identify mental health symptoms, offer recommendations for improving mental health and provide coping strategies for emotional distress based on the analysis of students' digital footprints or text conversations (Grové 2021).

Currently, some schools provide virtual counselling through AI‐driven chatbots that provide immediate support for students experiencing mild to moderate mental health issues (D'Alfonso 2020). Chatbots such as Woebot and Wysa provide practical strategies based on cognitive‐behavioural therapy (CBT) principles to enhance accessibility to mental health resources without the need for direct human intervention. Lee et al.'s (2020) study indicates that these chatbots can help alleviate symptoms of anxiety and depression by engaging students in structured conversations that promote self‐reflection and coping mechanisms. The benefits and criticisms of such chatbots will be discussed further below.

More recent work in the domain of non‐pharmacological/embodied digital therapies offers an interesting point of contrast to the present study. For example, Radanliev (2024) explores dance movement therapy (DMT) within extended reality or Metaverse environments as a non‐pharmacological intervention for anxiety, depression and other mental health conditions. That work emphasises immersive embodied, non‐verbal and non‐text interaction, movement and sensor feedback, while also highlighting challenges around data privacy, ethical consent and user security in metaverse deployment.

Compared with these immersive, movement‐based modalities, our study's focus on GenAI chatbot therapy engages a different dimension of digital mental health support, one mediated by text, dialogue and linguistic empathy rather than bodily enactment. Unlike DMT systems, chatbots require users to verbalise emotional states in text, which may privilege students more comfortable with verbal expression. Moreover, while both approaches surface concerns around privacy and trust, our participants emphasised issues of emotional authenticity, emotional misinterpretation and algorithmic over‐reliance, rather than sensor inaccuracies or real‐world embodiment mismatch. In this way, our findings complement the DMT/Metaverse literature by illuminating the particular affordances and limits of text‐based AI therapy in youth educational contexts.

### 2.3 Benefits and Challenges of Using AI Chatbots in Mental Health Care

AI chatbots can provide accessible, immediate care and their constant availability may be especially valuable for those facing barriers related to geography, cost, or stigma (D'Alfonso 2020). Chatbots can reduce the workload of mental health professionals by helping to handle routine interactions, freeing clinicians to focus on complex cases (Sweeney et al. 2021). This flexibility supports proactive mental health management and can facilitate early intervention through timely reminders and structured advice. Individuals may feel more comfortable sharing sensitive information with AI due to the anonymity and perceived lack of judgement. Research has shown that secondary school students who hesitate to seek help from human counsellors may find AI‐based tools appealing due to their ease of use and consistent, non‐judgemental responses (Lee et al. 2020). Additionally, chatbots that apply CBT strategies can reinforce positive health behaviours, making mental health support more comprehensive (D'Alfonso 2020; Sweeney et al. 2021).

However, AI chatbots have been criticised for their limited ability to fully comprehend and respond empathetically to complex emotions, impacting their role in responding to deep emotional needs (Sweeney et al. 2021). As an example, an AI chatbot for mental health support in schools was reported as lacking in ‘listening’ skills, moving too quickly into problem solving mode instead of communicating with users on their concerns (Grové 2021). Chan (2025) applied Grodniewicz and Hohol's three‐challenge framework—the Problem of a Confused Therapist, Problem of a Non‐Human Therapist and Problem of a Narrowly Intelligent Therapist, to analyse secondary students' perceptions of GenAI chatbots as potential therapeutic agents, revealing that students valued accessibility but were sceptical about AI's ability to provide genuine empathy, trust and adaptability. Trust in AI is a critical factor, yet overly human‐like chatbots may cause an ‘uncanny valley effect’—the discomfort or eerie feeling experienced when interacting with AI or robotic systems that appear almost, but not quite, human (Ciechanowski et al. 2019). In mental health contexts, this can impact the acceptance and effectiveness of AI‐driven therapy chatbots, where clients may find it unsettling and have difficulty forming a trusting therapeutic relationship with the tool.

Moreover, privacy concerns are particularly pronounced in both educational and mental health support settings. The collection of sensitive emotional and psychological data poses risks, as misuse or breaches can erode trust and deter individuals from using these tools. Additionally, clinical AI applications tend to lack transparency regarding the training of their large language models (King et al. 2023), and algorithmic bias in AI training can lead to inequitable responses that negatively affect marginalised groups (Khawaja and Bélisle‐Pipon 2023). Furthermore, sustaining long‐term interaction can be difficult if chatbot responses become repetitive (D'Alfonso 2020). Such inherent limitations of AI raise caution on the use of AI tools as standalone solutions within the healthcare sector.

There is also a risk that over‐reliance on AI chatbots may reduce motivation for individuals to seek human professionals, which compromises their care (Jain et al. 2024). AI therapy is still in an experimental stage and its capabilities are still in question; there may be ‘therapeutic misconceptions’ where users overestimate the chatbot's capabilities (Appelbaum et al. 1982). These misconceptions may occur when individuals overestimate an AI tool's capabilities for providing understanding, empathy or tailored care, whereas it is actually meant for data processing, diagnostics, or supplementary support (for recent reviews on AI chatbots and mental health, please see Frances 2025; Hua et al. 2025; Mayor 2025). This can lead to misplaced trust in AI systems that are not designed for direct clinical treatment, potentially delaying necessary human intervention and worsening conditions (Ciechanowski et al. 2019).

In Chan and Colloton's (2024) AI literacy framework, they highlight one essential component—‘AI affectiveness for human emotions’—which refers to the need to understand how AI can recognise, interpret and respond to human emotions. Humans must recognise the implications of AI mimicking human behaviours and interactions; for example, being aware that while AI can simulate emotionally‐sensitive responses, it does not genuinely experience or understand emotions. ‘Healing, in its essence, often transcends mere text or verbal communication. Meaningful connections, deep acceptance and empathy between individuals arise from a shared understanding of lived experiences, sometimes conveyed through simply being present with one another—something that an AI agent can never replicate’ (Tse 2026). This crucial understanding and awareness will allow individuals to leverage AI's capabilities without misplacing trust or forming unrealistic expectations, better navigating the ethical and social complexities of interacting with emotionally responsive AI systems.

## 3 Methodology

This study draws on the AI Literacy Framework proposed by Chan and Colloton (2024), particularly the dimension of AI affectiveness for human emotions, which examines how well users understand AI's capacity to simulate, but not feel or comprehend, human emotions. This framework helps contextualise students' perceptions of empathy, trust and therapeutic efficacy in AI chatbots. The concept of therapeutic misconception (Appelbaum et al. 1982) is also used to interpret students' misplaced expectations of AI as equivalent to human therapists. These frameworks together inform the design, analysis and interpretation of this study's findings.

A qualitative research design was utilised to explore secondary school students' perceptions toward the use of GenAI as virtual mental health therapists. The data collection was conducted as part of an AI literacy programme facilitated by the primary author and her team, with students participating remotely. The course spanned 10 h of online modules that featured practical training and activities, covering core topics such as AI concepts, prompt engineering and AI ethics. The main goals of the programme were for students to learn about the ethics, safety and responsibilities of using GenAI, and to achieve an intermediate literacy level in GenAI.

Data for this study were collected from one activity within an online AI literacy module. In this activity, students were asked a guiding question: ‘Would you open up to an AI chatbot therapist?’ To support their reflection, they first watched a short explanatory video on the role of GenAI in mental health support, followed by hands‐on engagement with GenAI chatbots (e.g., ChatGPT). Students tested the chatbot's responses to emotionally driven inputs, noting their impressions and reflecting critically on both the benefits and limitations of using AI for mental health support.

While no strict word limit was enforced, students were instructed to submit detailed written reflections addressing both advantages and concerns. All reflections were written in English. Convenience sampling was adopted, as data were collected from the course and participation in this study was voluntary. In total, 69 students from various secondary schools in Hong Kong participated, aged 13–17 years (mean age = 15). The participants included 35 females, 21 males and 13 students who did not indicate their gender. Ethical approval for the study was obtained from participants and their parents prior to data collection, and all submissions were anonymised before analysis.

### 3.1 Data Analysis

An inductive thematic analysis approach was adopted to explore students' perceptions, following the methodological guidelines outlined by Elo and Kyngäs (2008). This method was chosen to allow patterns and themes to emerge organically from the data without imposing preconceived categories. The analysis process involved several iterative stages to ensure rigor and trustworthiness.

The research team comprising two primary researchers and two trained research assistants began with independent, close readings of all 69 student reflections to develop a holistic understanding of the content and to enhance immersion in the data. During this familiarisation phase, attention was paid to initial impressions, tone and emotionally charged language that might indicate underlying concerns or values.

Next, open coding was applied to the data, with each team member independently identifying meaningful units of text (e.g., words, phrases or sentences) that captured key ideas, sentiments or perceptions related to the use of GenAI as a mental health therapist. Codes were not predefined but emerged directly from the content, ensuring sensitivity to participants' language and perspectives.

These initial codes were then compiled and compared across coders to assess consistency. Coding discrepancies were discussed and resolved through collaborative dialogue and consensus‐building. When disagreements could not be resolved through initial discussion, a senior researcher reviewed the data segment and provided input to ensure alignment with the broader analytic goals.

Codes were subsequently grouped into higher‐order categories and themes that captured the shared benefits and challenges perceived by students. This involved an iterative refinement process, in which preliminary themes were reviewed, renamed, merged or split based on their coherence, relevance and representativeness. The thematic structure was revised multiple times to ensure it accurately reflected the breadth and depth of student responses.

While no qualitative analysis software (e.g., NVivo or Atlas.ti) was used in this study, all coding and theme development were conducted using Microsoft Excel, with data organised into matrices to facilitate comparison, theme tracking and auditability. Memos and annotations were used to document analytic decisions throughout the process.

The final themes were categorised under two overarching domains: (Abd‐Alrazaq et al. 2021) Benefits of GenAI as School Therapists and (Appelbaum et al. 1982) Challenges and Risks of GenAI in School Mental Health Contexts. These themes formed the foundation for the Results and Discussion sections, providing insight into the nuanced perceptions held by secondary school students in Hong Kong.

## 4 Results

Out of the 69 students, 18 fully supported the idea of using AI as a mental health therapist, emphasising its accessibility, non‐judgemental nature and availability. Conversely, 28 students opposed the concept, citing concerns such as the lack of empathy, potential privacy risks and the inability of AI to replicate human connection. Sixteen stated they would consider using AI for trivial matters, such as venting or addressing minor emotional issues. Five students expressed uncertainty or mixed feelings, suggesting their stance would depend on future advancements or specific contexts, and two students did not explicitly state their position on the matter. These findings underscore the varied perspectives on AI's role in mental health, reflecting both optimism and reservations about its capabilities and limitations. Table 1 shows the benefits and challenges of GenAI as therapists.

**TABLE 1:** Benefits and challenges of GenAI Chatbot as school therapists.

Benefits of GenAI as therapists	Challenges of GenAI as therapists
Real time, accessibility and 24/7 availability	Avoidance of sensitive topics
2 Anonymity and non‐judgemental nature	2 Concerns about over‐reliance on AI
3 Cost‐effectiveness	3 Ethical, privacy and data security concerns
4 Consistent and unbiased support	4 Dependence on human connection for complex situations
5 Initial and supplementary support	5 Inability to detect non‐verbal cues
6 Educational and informational support	6 Lack of empathy and emotions
	7 Risk of inadequate or harmful responses

### 4.1 Benefits of GenAI as Therapists

The integration of GenAI as a therapeutic tool in secondary schools presents multifaceted benefits, as expressed by student voice and supported by current literature.

#### 4.1.1 Accessibility, Scalability and Real‐Time Interaction

The most frequently highlighted advantage of AI therapy chatbots was their constant availability. As one student noted, chatbot therapists represent ‘an innovative approach to mental health support, providing accessible and convenient resources for users. They are available 24/7, allowing individuals to seek help at any time’. Students also viewed the round‐the‐clock accessibility as ‘like having someone available when you need quick reassurance, without needing to book an appointment’, which is important as ‘incidents can happen at any time’ and ‘it can then give you advice or information in real time’. In school settings where stress and anxiety can spike outside regular school hours, this accessibility offers crucial emotional assistance for those who may need support during high‐pressure periods.

Additionally, students recognised that AI is scalable and ‘can handle multiple conversations simultaneously, addressing the scalability challenge faced by human therapists’ while still being able to ‘give you advice or information in real time’. This immediacy contrasts with traditional therapy where students may need to wait days or weeks for an appointment.

#### 4.1.2 Anonymity and Non‐Judgemental Nature

A recurring theme in students' reflections was a sense of security and safety provided by the anonymous nature of AI interactions, which encourages openness. As explained by students, ‘the anonymity makes me feel more comfortable opening up to an AI therapist’ and ‘allows us to explore our emotions without fear of judgement or shame from others’.

AI therapy also offers a unique advantage for those who find face‐to‐face interactions challenging. Students commented, ‘As an introvert, much energy is needed for me to socialise and have a deep talk with others’, and, ‘Some people do not feel comfortable [reaching out for help from others] … using ChatGPT helps them’. Moreover, using AI chatbots can alleviate the worry or guilt of burdening others with one's own issues, with one student writing that ‘chatting with a chatbot doesn't bother anyone, so people can discuss their problems freely even if the issue is trivial’. For those who experience anxiety in social or therapeutic settings, AI offers a non‐threatening way to communicate their feelings.

#### 4.1.3 Affordability

For many, financial constraints are a significant barrier to accessing traditional mental health services. AI's affordability was cited as an advantage, with one student highlighting that ‘you can use AI for free/at a lower cost than typical therapists. Traditional therapy can cost a lot for a one‐hour session … Some people may not be able to afford therapy’. Another student pointed out that such advantages ‘could democratise access, especially in underserved communities’, allowing more in‐need individuals to prioritise seeking mental health support.

#### 4.1.4 Consistent and Unbiased Support

One significant benefit of AI therapy chatbots is their perceived consistency and impartiality. Some students appreciated that AI chatbots do not ‘have mood swings or off days. They provide consistent responses’, and that they ‘allow us to talk to them every time and without any limitation … [and] can provide a lot of useful information to us, in order to solve our problems’. Moreover, some also preferred AI's perceived objectivity, with one stating that, ‘AI applications are … capable of analysing people's woes and personal troubles from an objective perspective, as well as provide impartial and practical advice for people to deal with negative emotions’.

#### 4.1.5 Initial and Supplementary Support

AI can help provide immediate relief as well as a stepping stone towards seeking professional help. Some participants viewed chatbots as a good outlet for ‘trivial matters, just to vent out some anger or frustration’ and to ‘have a little bit of psychological comfort’. Multiple students commented on the potential for AI to act as a bridge towards professional support, where it can be ‘a valuable resource for those who need support but face barriers to traditional therapy’ as well as a way to help some individuals ‘build up the courage to talk to someone’.

Many students expressed optimism regarding the potentials of AI as a mental health support tool. Some noted that, ‘With enough research and development, GenAI could be my therapist’, and ‘as AI advances, chatbots will improve. Natural language processing and emotional understanding will evolve’. However, others recognised GenAI's current limitations, with one student remarking, ‘AI is improving… [but] human therapists have more experience about how to comfort and communicate with people’. Students also acknowledged that while AI should not replace traditional therapy, it can act as a supplementary tool to bridge gaps between human interactions and ‘provide backup for users’.

#### 4.1.6 Educational and Informational Support

Students valued AI for informational guidance. One student commented, ‘ChatGPT can answer any questions I ask it. It can also solve lots of problems and can help me deal with my problems’. This access to a vast repertoire of information allows students to discover ‘valuable coping strategies and educational resources, making it a useful supplementary tool for managing mental health’.

### 4.2 Challenges of GenAI as Therapists

The analysis revealed several challenges associated with the use of GenAI as mental health tools in schools. Students highlighted issues ranging from emotional disconnects in AI interactions to concerns about data privacy and trustworthiness, impacting their willingness to engage with these technologies for mental health support.

#### 4.2.1 Avoidance of Sensitive Topics

One significant challenge highlighted by students is that AI tends to avoid or inadequately address sensitive topics. This is particularly alarming when discussing critical issues like suicidal ideation. A participant remarked, ‘When people talk about sensitive topics such as suiciding [sic], AI will avoid this topic and doesn't really answer you. It can be dangerous if we use it for therapy’. Another student shared that when they expressed their ‘emotions and heartbroken [sic], ChatGPT was advising me how to be happy. At first, I thought it's not that bad … However, soon after, I realise that whatever I said it would only give me positive reply and advice. That's not what I actually need!’

#### 4.2.2 Concerns About Over‐Reliance on AI

Students resonated with concerns that AI therapy chatbots may cause individuals to disregard professional support. As one student wrote, ‘Relying on AI could discourage people from seeking real human connection and professional help, which are crucial for effective therapy’. This reflects broader discussions regarding ethical usage of AI, with the caution that it should supplement rather than replace in‐person mental health services.

#### 4.2.3 Ethical, Privacy and Data Security Concerns

Students were apprehensive about data security, with some noting that they ‘would not feel comfortable opening up to an AI therapist because you're telling your personal information to AI, knowing what you share will be used to train the AI system doesn't sound very private’, and ‘privacy, data security, and the potential for misuse raise ethical questions. Can a chatbot truly keep our secrets?’ Others further raised questions regarding the ethical challenges and insufficient regulations, such as ‘If a client's information is leaked somehow, who shall be responsible for the damage…?’

A strong theme of *warranted distrust* emerged, rooted in the scepticism that students felt about the safety and ethical handling of their data. As students pointed out, ‘Even for human therapists, there are many regulations to safeguard the client's privacy. There are currently no laws against AI exploiting human needs and emotions’, reflecting legitimate concerns that AI, without stringent regulations, poses numerous risks of data safety and misuse.

#### 4.2.4 Dependence on Human Connection for Complex Situations

Students repeatedly expressed that human therapists' ability to empathise and adapt to complex emotional states is still unmatched by AI. As one student wrote, ‘I personally believe that a therapist cannot be replaced by AI… the interpersonal relationship [makes] the clients feel safer and more comfortable’.

#### 4.2.5 Inability to Detect Non‐Verbal Cues

AI's current inability to interpret non‐verbal communication, such as body language and facial expressions, is a critical limitation. Students commented that ‘a significant part of therapy involves body language, eye contact and other subtle cues… ChatGPT lacks this capability, which limits its effectiveness’, as these cues are essential for recognising subtleties and nuances in a person's emotional state. Moreover, students acknowledged that even if GenAI becomes advanced enough to interpret these cues, there are many contextual factors that the student user may have the burden of explaining and providing, such as their background, culture, family status and relationships with friends, teachers and the school.

#### 4.2.6 Lack of Empathy and Emotions

Despite optimism for technological progress, students were aware of AI's current and foreseeable limitations. Some remarked that ‘although AI therapists [can] be trained to be more human‐like, we aren't sure that it will be able to imitate human empathy … or understand our emotions’ and ‘ChatGPT cannot detect emotions and feelings of human accurately’, highlighting that even if AI can simulate empathy through well‐trained responses, it lacks the intrinsic human qualities that contribute to genuine emotional connection and empathetic understanding. Similarly, others noted that ‘therapists in the real world do their job better as they may have experiences similar to your own, and [are] living life just like you’, highlighting the sentiment that no amount of programming can replace the inherent human ability to empathise based on shared experiences, making AI feel detached and superficial in its support.

Another recurring issue highlighted by participants was that AI may deliver superficial advice that fails to address deeper emotional needs, similar to its avoidance of sensitive topics. One student aptly asked, ‘Can [AI] correctly identify the hidden emotions that is beyond what the person types in the chat?’ This limitation calls for cautious integration to ensure that AI supports, but does not replace traditional human‐centred care, and the need for students to develop AI literacy.

#### 4.2.7 Risk of Inadequate or Harmful Responses

A risk frequently cited by students is the potential for AI to provide incorrect or insensitive advice which can be harmful to users, briefly mentioned above as well. Students commented that ‘There is always a chance that the bot will say the wrong thing and make matters worse’, and ‘If you were anxious about something and the chatbot used harmful words, it will make you even more anxious’. Students recognised that AI has not ‘been tested about its effectiveness regarding therapy’, understanding how easily AI could exacerbate a user's condition if not equipped with precise and empathetic programming.

## 5 Discussion

Students' responses in this study reflect findings in the existing literature, highlighting both the opportunities and limitations of AI tools in mental health services. Researchers such as Zidaru et al. (2021) and Elyoseph and Levkovich (2023) emphasise the advantages of AI's continuous availability and real‐time responsiveness, aligning with participants' appreciation of its accessibility and non‐judgemental nature. The sense of safety that students feel resonates with what Oghenekaro and Okoro (2024) highlight as the secure and anonymous space AI offers, encouraging users to open up without fear of judgement.

The findings of this study extend current research on AI‐based mental health support by demonstrating how secondary students negotiate both the promise and limitations of GenAI in school counselling contexts. Recent reviews (Hua et al. 2025; Mayor 2025) indicate that large language model chatbots are shifting from informational tools to emotionally responsive systems; however, the present results show that students remain cautious about equating simulated empathy with genuine therapeutic presence. While prior work has highlighted the accessibility and cost‐effectiveness of AI counselling for youth (Labadze et al. 2023; Oghenekaro and Okoro 2024), our participants emphasised a different value: AI as a low‐stakes, low‐judgement entry point into help‐seeking, particularly for students reluctant to approach a human counsellor. This extends earlier findings by Lee et al. (2020), suggesting that for adolescents in high‐pressure school environments, GenAI may operate as a bridge rather than an endpoint for mental health support.

Whereas previous studies have reported positive clinical or behavioural outcomes from chatbots such as Woebot or Wysa, the present study highlights limitations more specific to school settings. For instance, students' concerns about privacy and emotional safety parallel ethical critiques raised by Khawaja and Bélisle‐Pipon (2023) and King et al. (2023), but our findings add nuance by showing how these concerns are amplified when data are shared within institutional ecosystems such as schools. In contrast to optimistic claims that GenAI may eventually ‘replace’ elements of human counselling (Frances 2025), students in this study consistently positioned AI as supplementary and conditional, valuable for initial regulation of anxiety or clarification of thoughts, but insufficient for deeper or crisis‐related support. This aligns more closely with Sharma et al.'s (2023) Human‐in‐the‐Loop model, suggesting that meaningful impact emerges when AI is embedded within a relational, supervised system rather than offered as a stand‐alone therapist.

To interpret these findings more deeply, this study draws on Chan and Colloton's (2024) AI Literacy Framework, specifically its component of *AI affectiveness for human emotions*, and the concept of therapeutic misconception (Appelbaum et al. 1982). The AI Literacy Framework emphasises users' understanding of how AI recognises and simulates emotions without possessing genuine empathy. Students' reflections revealed varied levels of affective AI literacy: some recognised that AI responses were formulaic and emotionally shallow, while others anthropomorphised the technology, believing it could genuinely ‘understand’ or ‘comfort’ them. This overestimation of AI's emotional capabilities reflects therapeutic misconceptions, where users conflate algorithmic empathy with human understanding. By framing the findings through these theoretical lenses, the study situates students' mixed reactions within the broader discourse on human–AI emotional interaction, revealing how emotional misunderstanding and limited AI literacy shape the formation of trust (Choung et al. 2023), comfort and scepticism in AI‐mediated mental health contexts.

Furthermore, the findings must be considered within the sociocultural context of Hong Kong. Secondary students in Hong Kong face intense academic pressures, particularly related to the high‐stakes DSE examination system, and often experience stigma surrounding mental health (Austin et al. 2025). These cultural factors contribute to students' appreciation for AI's anonymity and constant availability while simultaneously amplifying their concerns about privacy, emotional authenticity and data misuse. In a densely digital environment where smartphone use is ubiquitous, Hong Kong students are technologically literate but emotionally cautious, seeking balance between convenience and trust. Understanding this local context is essential to designing AI tools that are not only functional but culturally sensitive and emotionally appropriate.

To address these risks, researchers like Sharma et al. (2023) advocate for Human‐in‐the‐Loop AI, ensuring that AI technologies supplement rather than replace human therapists. Correspondingly, Elyoseph and Levkovich (2023) suggest that AI's real‐time responses can help users manage situational stress, serving as a gateway to comprehensive care from human professionals. Finally, the ethical implications of integrating AI into mental health contexts, including data security and liability, also remain critical, with Lee et al. (2020) emphasising the importance of robust oversight and clear guidelines to safeguard users.

### 5.1 Understanding AI Affectiveness for Human Emotions

The students' reflections in this study highlight varied levels of understanding in regard to Chan and Colloton's (2024) *AI affectiveness for human emotions*. While some demonstrated an awareness of the limitations of AI in replicating human empathy, others overestimated its capabilities, highlighting gaps in their AI literacy. Students' interactions with GenAI reveal the importance of developing educational programmes that emphasise not just how AI operates but also how it aligns with or diverges from human emotional intelligence.

A particularly compelling insight was how some students felt disconnection when interacting with AI, describing responses as ‘cold and logical’. This affirms the *uncanny valley effect* as described by Ciechanowski et al. (2019), where students expressed discomfort when AI attempted to emulate human empathy and human interaction. Educating students about why this effect occurs and how AI‐generated empathy differs from human empathy is also essential for enhancing their AI literacy, which in turn will empower them to engage with GenAI in more informed and realistic ways.

### 5.2 Therapeutic Misconception and Over‐Reliance

Therapeutic misconceptions (Appelbaum et al. 1982), where users overestimate an AI's ability to provide comprehensive emotional support, emerged as a theme among students' responses. This highlights the potential risks associated with relying on AI for mental health support, as such misconceptions can result in misplaced trust. Some students believed that because GenAI could ‘generate more suitable advice’, it was capable of offering therapy akin to human professionals; this belief can lead to an overreliance on AI and delay in seeking human intervention when needed, which could be detrimental to emotional well‐being. Additionally, some students already perceive GenAI to be highly believable. However, AI relies on algorithmic models that can produce inaccurate or even misleading advice (Chan and Colloton 2024; Khawaja and Bélisle‐Pipon 2023; Lee et al. 2020), lacking true understanding or reliability despite appearing capable of understanding emotions.

Developing AI literacy that clearly delineates the strengths and limitations of GenAI, particularly its affective capabilities, can help mitigate therapeutic misconceptions. Educators should ensure that students understand that AI's responses are based on data and algorithmic patterns, not lived experience or true empathy. This knowledge will help students use GenAI more responsibly, seeking human counselling when deeper emotional issues arise.

### 5.3 Warranted Distrust and Ethical Concerns

Students' responses highlighted warranted distrust, particularly around data privacy and ethical handling of sensitive information. They had concerns regarding their personal data being used to train AI models, compromising privacy. Such distrust is an essential part of AI literacy, as the recognition of ethical dimensions of AI interactions (Choung et al. 2023) allows students to approach AI tools with a balanced scepticism, using them effectively while being mindful of potential risks. Continuing to integrate discussions on privacy, data use and the regulatory landscape into AI education can equip students with the knowledge to make informed decisions about their use of GenAI. This aligns with fostering responsible AI usage, where students feel empowered to engage with technology while maintaining a critical view of its limitations and risks.

### 5.4 Balancing Optimism and Realistic Expectations

Despite their concerns, students also expressed optimism about future advancements in AI technology. Some believed that GenAI can eventually become a more effective tool for initial mental health support. This hopeful outlook reflects their understanding of AI's potential but must be balanced with realistic expectations fostered through improved AI literacy. Teaching students to appreciate AI's real‐time, accessible nature while understanding that it cannot replace the depth of human interaction is crucial for promoting healthy use. Educating students on these limitations further ties back to the Chan and Colloton's (2024) component of *AI affectiveness for human emotions* within their AI literacy framework.

These findings also advance the two frameworks guiding this study. First, through Chan and Colloton's (2024) AI literacy framework, students demonstrated uneven development of *affective AI literacy*: some recognised the programmed nature of chatbot ‘empathy’, while others anthropomorphised responses, assuming emotional understanding where none existed. This indicates that affective literacy is not merely a knowledge gap but a *psychosocial skill* requiring explicit instruction. Second, applying Appelbaum et al.'s (1982) concept of therapeutic misconception reveals how students may misinterpret algorithmic fluency as therapeutic competence. Our data clarify that misconceptions arise not only from technological opacity, as past literature suggests, but also from unmet emotional needs within school systems, making AI appear more capable than intended. Thus, the contribution of this study lies in positioning emotional misinterpretation as a *literacy problem rather than a technical failure*, offering new direction for research and practice in youth mental health and AI. These dynamics must also be read through the Hong Kong educational context, where academic pressure, stigma and collectivist expectations shape help‐seeking behaviour (Austin et al. 2025). The appeal of anonymous, judgement‐free AI support may therefore reflect cultural hesitancy toward emotional disclosure rather than confidence in AI capability. This extends Brown and Halpern's (2021) argument that chatbot acceptance is socially negotiated, and suggests that emotional safety, rather than technological advancement, may be the primary driver of adoption in schools.

## 6 Conclusion

The integration of GenAI as a tool for mental health support in educational settings is both a promising opportunity and a complex challenge. This paper highlights the multifaceted perspectives of secondary school students who have had first‐hand experiences with GenAI, emphasising how their AI literacy influences their perceptions and engagement with this technology. While students recognised benefits such as accessibility, real‐time support and non‐judgemental interactions, they also pointed out substantial limitations, including emotional disconnect, ethical concerns and the risk of over‐reliance. Their insights align with the need for balanced use, where AI serves as a supplement rather than a replacement for human interaction.

The exploration underscored the importance of enhancing user AI literacy, particularly fostering an understanding of AI's capabilities and limitations when responding to emotional content and cues. By teaching students to critically evaluate AI's affective responses, manage therapeutic misconceptions and navigate warranted distrust, educational programmes can empower young users to engage responsibly with GenAI. Moving forward, the reflections of students in this study serve as a reminder that while technology continues to advance, it must be integrated thoughtfully to enhance traditional mental health services.

### 6.1 Practical Implications for Policy and School Practice

The findings underscore the need for educational policy and school systems to take a proactive role in guiding safe and ethical engagement with GenAI‐based mental health tools. At the policy level, education authorities should establish clear AI governance guidelines for school‐based wellbeing technologies, including data protection standards, informed consent procedures and teacher oversight requirements. At the school level, AI literacy should be embedded into the personal, social and health education curriculum to equip students with the knowledge to distinguish simulated empathy from genuine emotional understanding. Schools can also implement Human‐in‐the‐Loop systems, where counsellors supervise AI chatbots to ensure responsible and supportive use.

Research and evaluation should be embedded throughout the process, drawing on locally responsive data that reflects the collectivist orientations of Hong Kong communities, for example, the strong influence of family and parental control over students' use of AI chatbots. In some cases, parents may hold misconceptions, such as assuming that anything involving computers is inherently academically smart and beneficial (Tse and Ng 2014).

Moreover, teacher professional development programmes should focus on AI feedback literacy and AI ethics awareness, enabling educators to recognise signs of over‐reliance on AI or student distress triggered by AI interactions. Finally, local developers should be encouraged to co‐design culturally sensitive AI support tools in partnership with Hong Kong educators and psychologists, ensuring that the technology resonates with students' lived experiences and sociocultural values (see Table 2).

**TABLE 2:** Practical implications for policy and school practice on the intersection of AI and student well‐being.

Practical implications for policy and school practice on the intersection of ai and student well‐being
*AI Literacy Integration in Curriculum* AI literacy, particularly its affective dimension should be embedded into Hong Kong's life education and digital citizenship curricula. Students must be explicitly taught the limits of AI empathy and the ethical considerations of using GenAI for emotional or psychological purposes.
*Human‐in‐the‐Loop (HITL) Safeguards* Schools adopting AI wellbeing tools should employ HITL systems in which human counsellors or teachers monitor chatbot interactions. AI should serve as an early support mechanism, not as a substitute for professional care.
*Teacher Professional Development* Teacher training should include AI feedback literacy, enabling educators to recognise student over‐reliance on AI or signs of emotional detachment resulting from AI use. This will help teachers guide students toward appropriate human counselling when needed.
*Policy Guidelines for Ethical AI Use in Schools* Education authorities should develop regulatory frameworks and school‐based AI guidelines that clarify data ownership, privacy and informed consent. Transparent data management policies are critical to reducing students' warranted distrust of AI systems.
*Culturally Responsive Design of AI Tools* Collaboration between AI developers, educators and psychologists in Hong Kong should ensure that AI mental health chatbots reflect local language nuances, values and cultural norms, making them more relatable and ethically aligned with student needs.

## 7 Limitations and Future Directions

This study had several limitations. First, the sample was limited to secondary school students within a specific region, which may not fully represent the diverse range of experiences and cultural contexts that could influence perceptions of GenAI. Additionally, the focus on students with prior exposure to AI tools may mean that findings are more reflective of tech‐literate individuals rather than the general student population. Future research should expand to include a more diverse range of participants across different regions and levels of AI familiarity.

The study was also conducted during a one‐time AI literacy training programme and did not consider changes in perceptions and behaviours with sustained exposure to GenAI. Longitudinal studies should be conducted to observe changes in students' perceptions and engagement with GenAI as technology evolves and AI literacy improves. Finally, the qualitative nature of the research also limits transferability of findings. The integration of quantitative approaches, such as surveys and structured observations, could enable future research to explore the perceptions and user behaviours of a wider student population concerning GenAI in school mental health services.

## Author Contributions

**Cecilia K. Y. Chan:** conceptualisation ideas, formulation or evolution of overarching research goals and aims, methodology, development or design of methodology, formal analysis, writing – original and final drafts, writing – review and editing. **Samson Tse:** writing – original and final drafts, writing – review and editing. All authors have given final approval of the version of the manuscript to be published.

## Funding

This study was funded by UGC FITE Funding and HKU Seed Funding CRF.

## Ethics Statement

This study was conducted in full compliance with institutional and international ethical standards for research involving human participants. Ethical approval was obtained from the institutional research committee at the University of Hong Kong (ethical approval no.: EA250285), and all procedures adhered to the principles outlined in the 1964 Helsinki Declaration and its later amendments. Participation was entirely voluntary. All written reflections were anonymised at the point of submission to ensure the confidentiality and privacy of participants. No personally identifiable information was collected or retained throughout the study. Participants were informed of the study's purpose, their right to withdraw at any time without consequence, and how their data would be used. All data were securely stored and accessed only by authorised members of the research team. No deception or potentially harmful procedures were involved in the study.

## Consent

Informed consent was obtained from parents of underage participants included in the study; assent was obtained from underage participants.

## Conflicts of Interest

The authors declare no conflicts of interest.

## Data Availability

The data that support the findings of this study are available on request from the corresponding author. The data are not publicly available due to privacy or ethical restrictions.
